# Administration of an antibody against apoptosis inhibitor of macrophage prevents aortic aneurysm progression in mice

**DOI:** 10.1038/s41598-024-66791-7

**Published:** 2024-07-10

**Authors:** Taro Fujii, Aika Yamawaki-Ogata, Sachie Terazawa, Yuji Narita, Masato Mutsuga

**Affiliations:** Department of Cardiac Surgery, Nagoya University Graduate School of Medicine, 65 Tsurumaicho Showa, Nagoya, Aichi 466-8550 Japan

**Keywords:** Aortic aneurysm, Apoptosis inhibitor of macrophage, Macrophage, Inflammation, Aortic diseases, Experimental models of disease

## Abstract

Apoptosis inhibitor of macrophage (AIM) is known to induce apoptosis resistance in macrophages and to exacerbate chronic inflammation, leading to arteriosclerosis. The role of AIM in aortic aneurysm (AA) remains unknown. This study examined the effects of an anti-AIM antibody in preventing AA formation and progression. In apolipoprotein E-deficient mice, AA was induced by subcutaneous angiotensin II infusion. Mice were randomly divided into two groups: (i) AIM group; weekly anti-murine AIM monoclonal antibody injection (n = 10), and (ii) IgG group; anti-murine IgG antibody injection as control (n = 14). The AIM group, compared with the IgG group, exhibited reduced AA enlargement (aortic diameter at 4 weeks: 2.1 vs. 2.7 mm, respectively, p = 0.012); decreased loss of elastic lamellae construction; reduced expression levels of IL-6, TNF-α, and MCP-1; decreased numbers of AIM-positive cells and inflammatory M1 macrophages (AIM: 1.4 vs. 8.0%, respectively, p = 0.004; M1 macrophages: 24.5 vs. 55.7%, respectively, p = 0.017); and higher expression of caspase-3 in the aortic wall (22.8 vs. 10.5%, respectively, p = 0.019). Our results suggest that administration of an anti-AIM antibody mitigated AA progression by alleviating inflammation and promoting M1 macrophage apoptosis.

## Introduction

Aortic aneurysm (AA) is characterized by continuous aortic enlargement due to chronic inflammation and arteriosclerosis, and is extremely life threatening after rupture. Surgical treatment is currently the standard of care. Open surgery completely excises AA and replaces it with a prosthetic vascular graft. However, it is enormously invasive and is associated with high morbidity and mortality, especially in cases of thoracic and thoracoabdominal AA^1^. Endovascular stent-graft repair is a less invasive, catheter-based procedure. However, secondary interventions are sometimes required due to challenges such as anatomical restrictions, graft migration, and endoleaks^2^. Both treatment approaches are implemented once AA reaches an indicated size, and early interventions are not currently recommended regardless of the surgical method^3^. A variety of pharmacological agents have been used to control AA progression, including antihypertensive drugs, antiplatelet drugs, and hypolipidemic drugs, but no specific agents have been compelling enough to bring about innovative changes in AA therapeutic strategies^4^.

The underlying pathophysiology of AA involves the destruction of the extracellular matrix (ECM) of the aortic wall, where chronic inflammation promotes aneurysm formation and progression, eventually leading to catastrophic rupture. Various inflammatory cytokines and chemokines, such as interleukin (IL)-1β, IL-6, tumor necrosis factor (TNF)-α, and monocyte chemotactic protein (MCP)-1, promote this destructive process through inflammatory cell infiltration, matrix metalloproteinase (MMP) secretion, and macrophage recruitment^5^. Recent studies have revealed that macrophages with the pro-inflammatory phenotype, namely M1 macrophages, play a crucial role in the production of cytokines that exacerbate chronic inflammation^6,7^. Further, M1 macrophages induce the activation of MMPs, which leads to the degradation of ECM components such as collagen and elastin^6^. In our previous research we sought to establish a new medical therapy that influences these pathological mechanisms by regulating macrophages. For example, in a study using mesenchymal stem cells (MSCs), we demonstrated that MSCs exerted a paracrine effect on AA, specifically by decreasing the number of M1 macrophages and increasing that of anti-inflammatory M2 macrophages^8^.

To suppress the chronic inflammatory effects of macrophages in AA, we have focused on the ability of apoptosis inhibitor of macrophage (AIM) to inhibit macrophage apoptosis^9,10^. AIM is a secreted protein specifically produced in macrophages, and was first discovered by Miyazaki et al^9^. This protein, also known as CD5L, is a member of the scavenger receptor cysteine-rich domain superfamily^11,12^. AIM not only inhibits macrophage apoptosis but also increases M1 macrophage recruitment to inflammatory tissues by enhancing MCP-1 expression^13^. A recent study demonstrated that an anti-AIM antibody decreased inflammatory responses and increased survival rates in an experimental sepsis model^14^. In atherosclerotic lesions, AIM facilitates macrophage survival, while loss of AIM increases macrophage apoptosis^15^. Although AIM may be a critical regulatory factor that modulates aneurysmal progression, few studies have assessed its efficacy in treating AA.

We hypothesized that administering an anti-AIM antibody could reduce the number of M1 macrophages and suppress ECM degradation and eventual AA formation and development. This is the first study to examine the potential effects and underlying molecular mechanisms of blocking AIM in AA.

## Results

### Incidence and progression of AA

**Figure 1:**
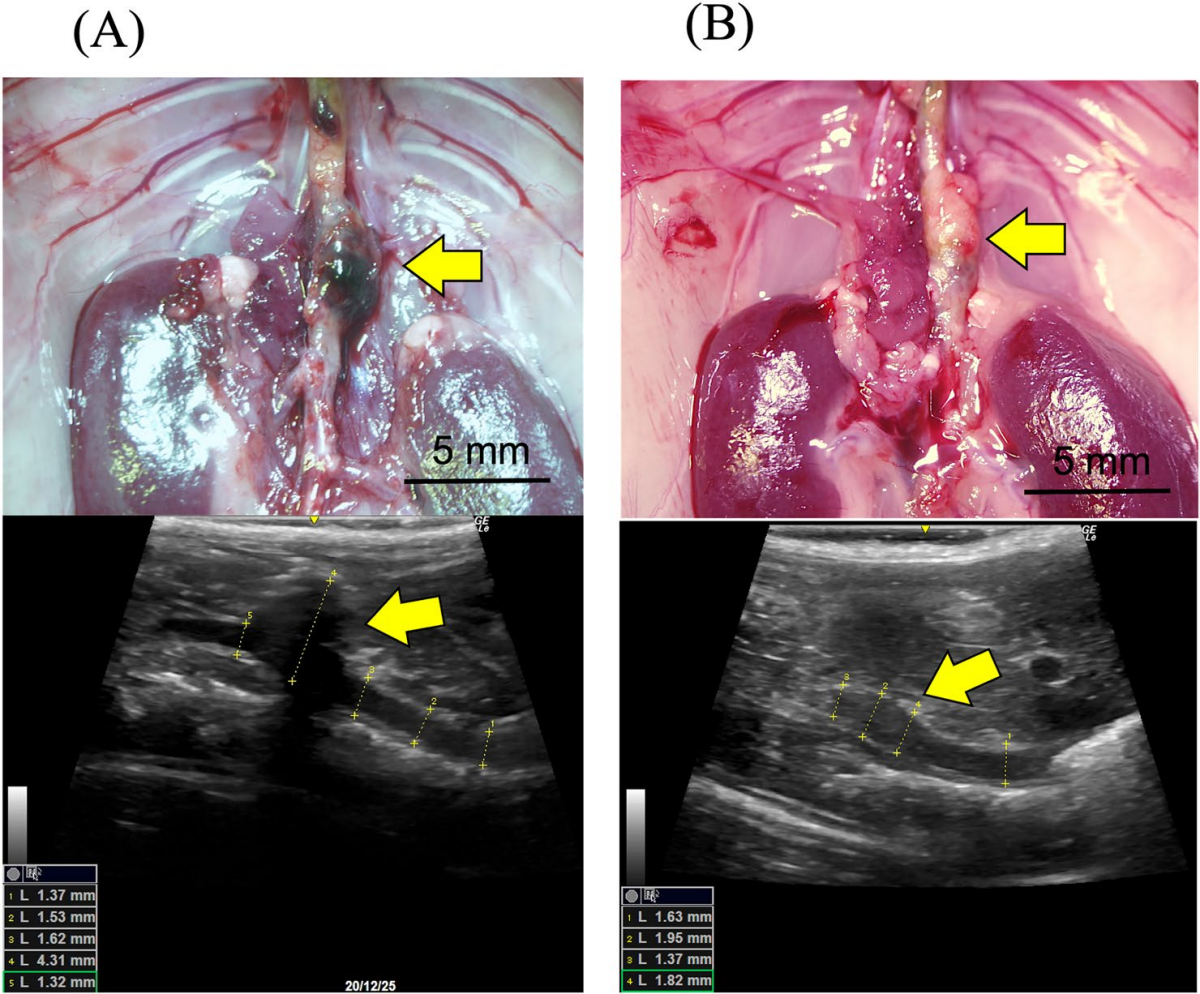
Representative microscopic and echography images of the aorta in each group. (**A**) 4 weeks in the IgG group, and (**B**) 4 weeks in the AIM group. Arrows indicate AAs. Scale bars = 5 mm.

**Figure 2:**
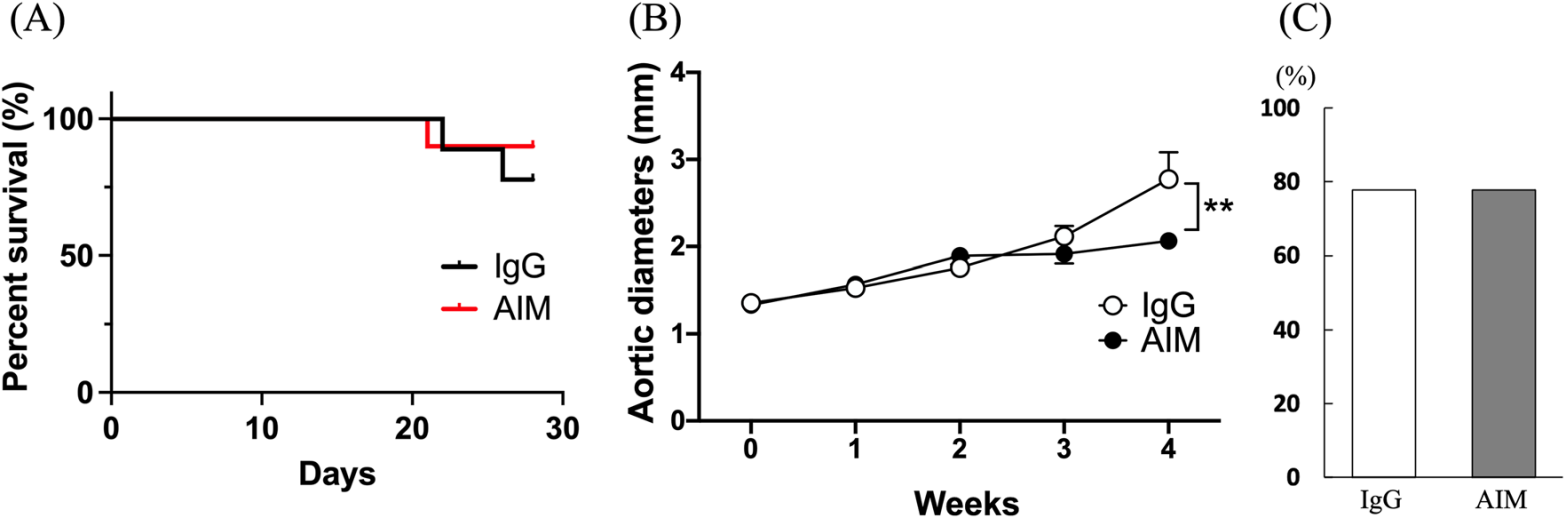
(**A**) Kaplan–Meier survival curves show the survival rates in the two groups. (**B**) Aortic diameters over time were assessed by two-way ANOVA followed by Tukey’s multiple comparisons test. Data are means ± SEM. **p < 0.01 vs IgG. (**C**) Fisher’s exact test was used to assess the incidence of AA.

There was one death in the AIM group, and two in the IgG group. Twenty-one animals survived for 4 weeks and were sacrificed to examine induced AA (Fig. 1). Three animals in the IgG group were omitted due to failure of AA formation, defined by less than a 1.3-fold increase in aortic diameter, in addition to little or no calcification or aneurysmal changes in the aortic wall. There was no significant difference in the survival rate between the two groups (Fig. 2A). Although the causes of death were undetermined in all cases, there was no evidence of aneurysmal rupture under laparotomy. Consequently, the analyses were performed with nine animals in each group. As shown in Fig. 2B, the aortic diameter at 4 weeks, as assessed by echography, was significantly smaller in the AIM group than in the IgG group (2.06 ± 0.06 vs. 2.73 ± 0.31 mm, respectively, p = 0.0012). However, there was no significant difference in the incidence of AA at 4 weeks (Fig. 2C).

### Elastic lamellae

**Figure 3:**
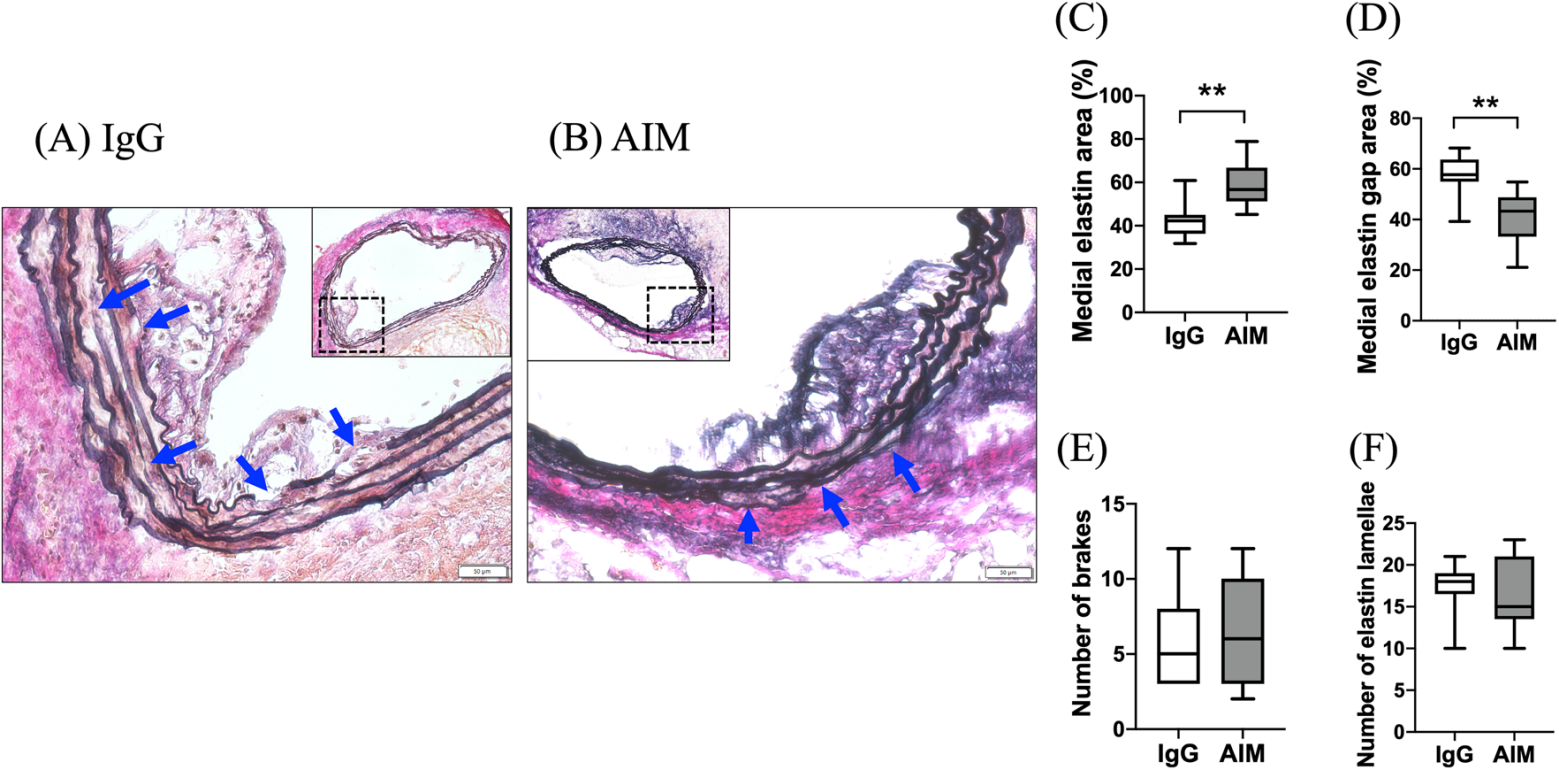
(**A**,**B**) EVG staining shows destruction of the elastic lamellae in the aortic wall at 8 weeks. The blue arrows indicate breaks in the elastic lamellae. Scale bars = 50 μm. (**C**,**D**) The medial and medial elastin gap areas are significantly different between groups. (**E**,**F**) The numbers of breaks and elastin lamellae are not significantly different. Mann–Whitney test, **p < 0.01.

EVG staining showed destruction of the elastic lamellae at 8 weeks (Fig. 3A, B). The percent ratio of the medial elastin area to the total medial tissue area was significantly higher in the AIM group than in the IgG group (Fig. 3C, 58.7 vs. 47.7%, respectively, p = 0.033). In contrast, the percent ratio of the medial elastin gap area to the total medial tissue area, representing broken elastic lamellae, was significantly lower in the AIM group (Fig. 3D, 43.3 vs. 57.7%, respectively, p = 0.0019). However, the numbers of breaks and elastic lamellae were not significantly different between the groups (Fig. 3E, F).

### Protein expression and enzymatic activity

**Figure 4:**
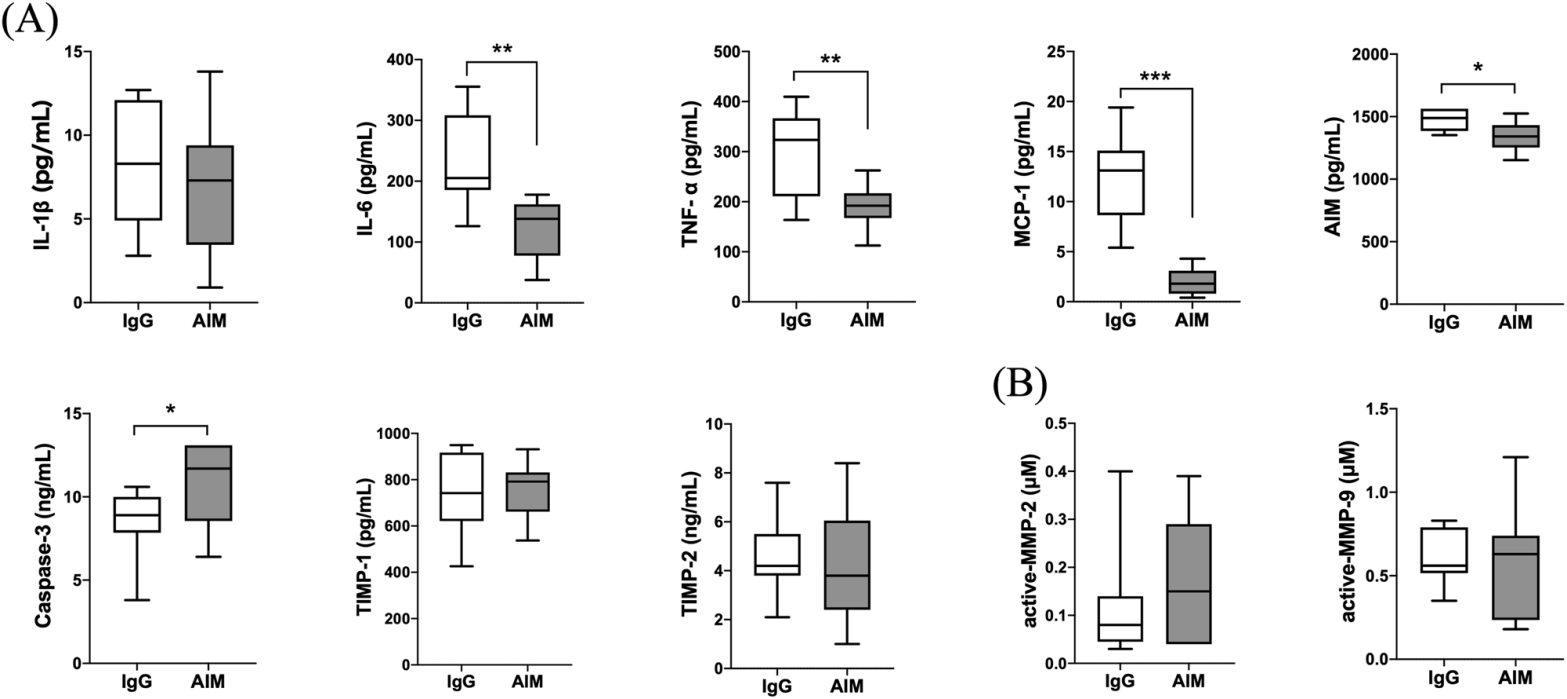
Quantitative analysis of protein expression levels in AA. (**A**) ELISA analysis of inflammatory cytokines (IL-1β, IL-6, TNF-α), a chemokine (MCP-1), caspase-3, and TIMPs (TIMP-1, TIMP-2). (**B**) Measurement of active MMP-2 and -9. Data are means ± SEM. *p < 0.05, **p < 0.01, and ***p < 0.001 assessed by the Mann–Whitney *U* test.

As shown in Fig. 4A, protein expression levels in the AIM group, in comparison with the IgG group, were lower at 4 weeks for IL-6 (124.2 ± 16.0 vs. 249.1 ± 32.0 pg/ml, respectively, p = 0.007), TNF-α (190.8 ± 13.2 vs. 319.0 ± 30.2 pg/ml, respectively, p = 0.004), MCP-1 (2.0 ± 0.4 vs. 13.8 ± 1.2 pg/ml, respectively, p < 0.001), and AIM (1347.0 ± 37.3 vs. 1489.7 ± 31.4 pg/ml, respectively, p = 0.013), and were higher for caspase-3 (22.8 vs. 10.5%, respectively, p = 0.019), while there was no statistical difference between the two groups in the expression levels of IL-1β, TIMP-1, or TIMP-2. Zymographic analysis of MMP-2 and -9 activity levels showed no significant differences at 4 weeks (Fig. 4B).

### Immunofluorescence staining

**Figure 5:**
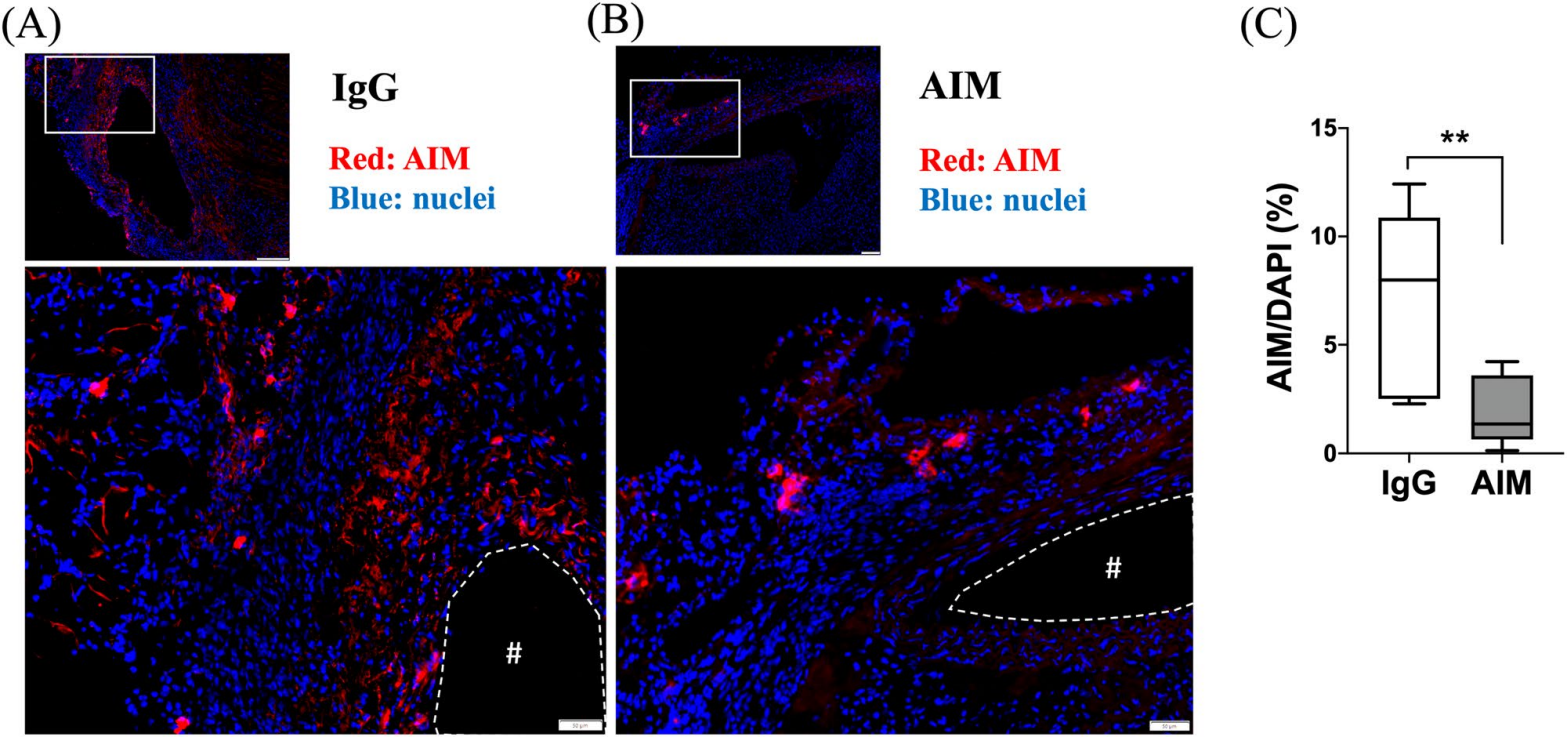
AIM distribution. (**A**,**B**) Representative images of immunofluorescence staining for AIM in the IgG and AIM groups. AIM (red) and cell nuclei (blue). Scale bars = 50 μm. (**C**) The ratio of the AIM-positive area to the DAPI-positive area, expressed as a percentage. Data are means ± SEM. **p < 0.01 assessed by the Mann–Whitney *U* test. The “#” symbols enclosed by dashed lines indicate the vessel lumens.

**Figure 6:**
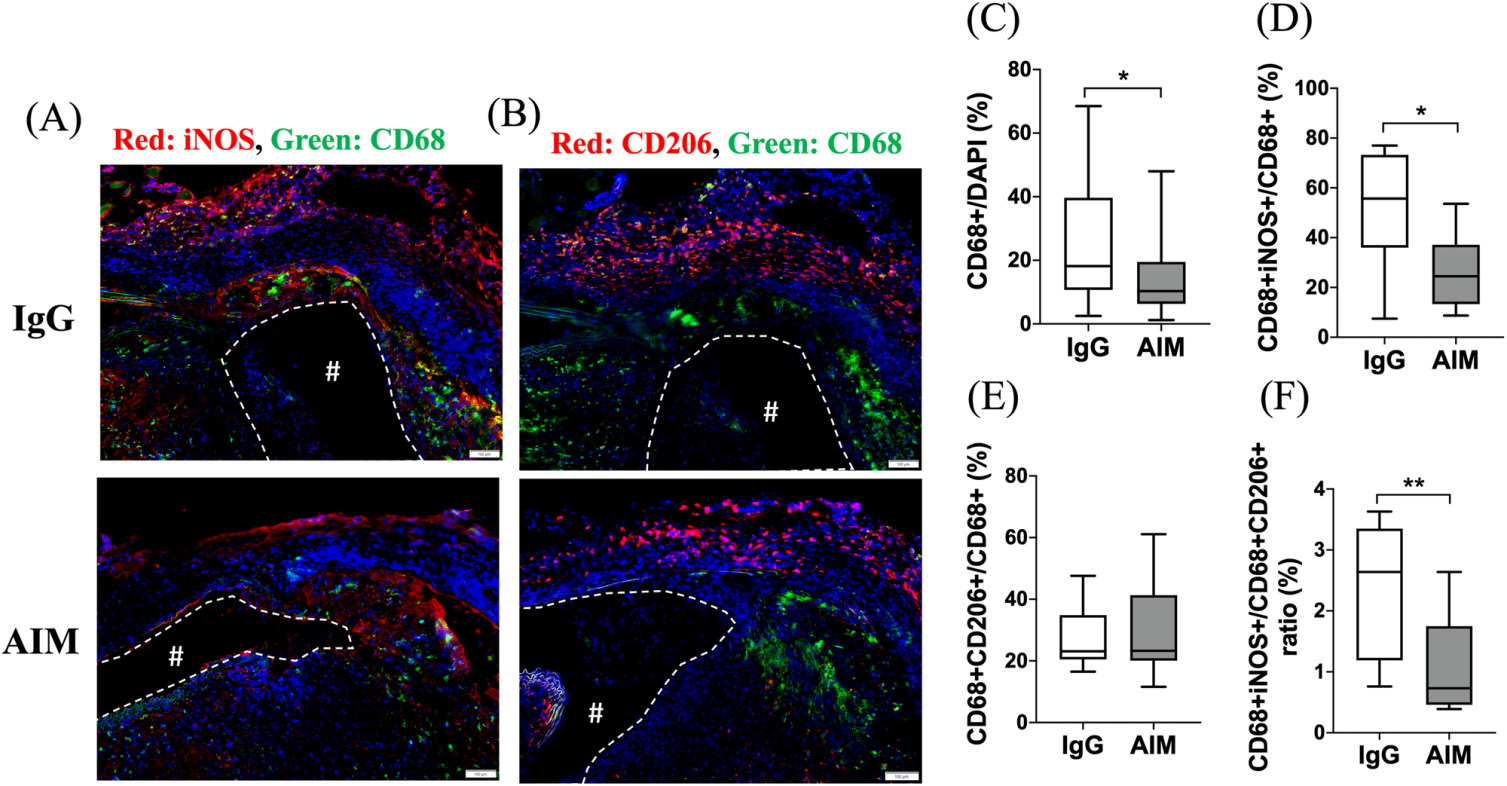
Macrophage polarization. (**A**) iNOS (red) and CD68 (green); (**B**) CD206 (red), CD68 (green), and cell nuclei (blue). Scale bars = 100 μm. (**C**) The percentage of CD68 + cells to DAPI + cells. (**D**,**E**) The percentage of the CD68 + population consisting of iNOS + /CD206 + cells. (**F**) The percent ratio of CD206 + cells to iNOS + cells in the CD68 + population. Data are means ± SEM. *p < 0.05 and **p < 0.01 assessed by the Mann–Whitney *U* test. iNOS + /CD68 + cells indicate M1 macrophages, while CD206 + /CD68 + cells indicate M2 macrophages. The “#” symbols enclosed by dashed lines indicate the vessel lumens.

**Figure 7:**
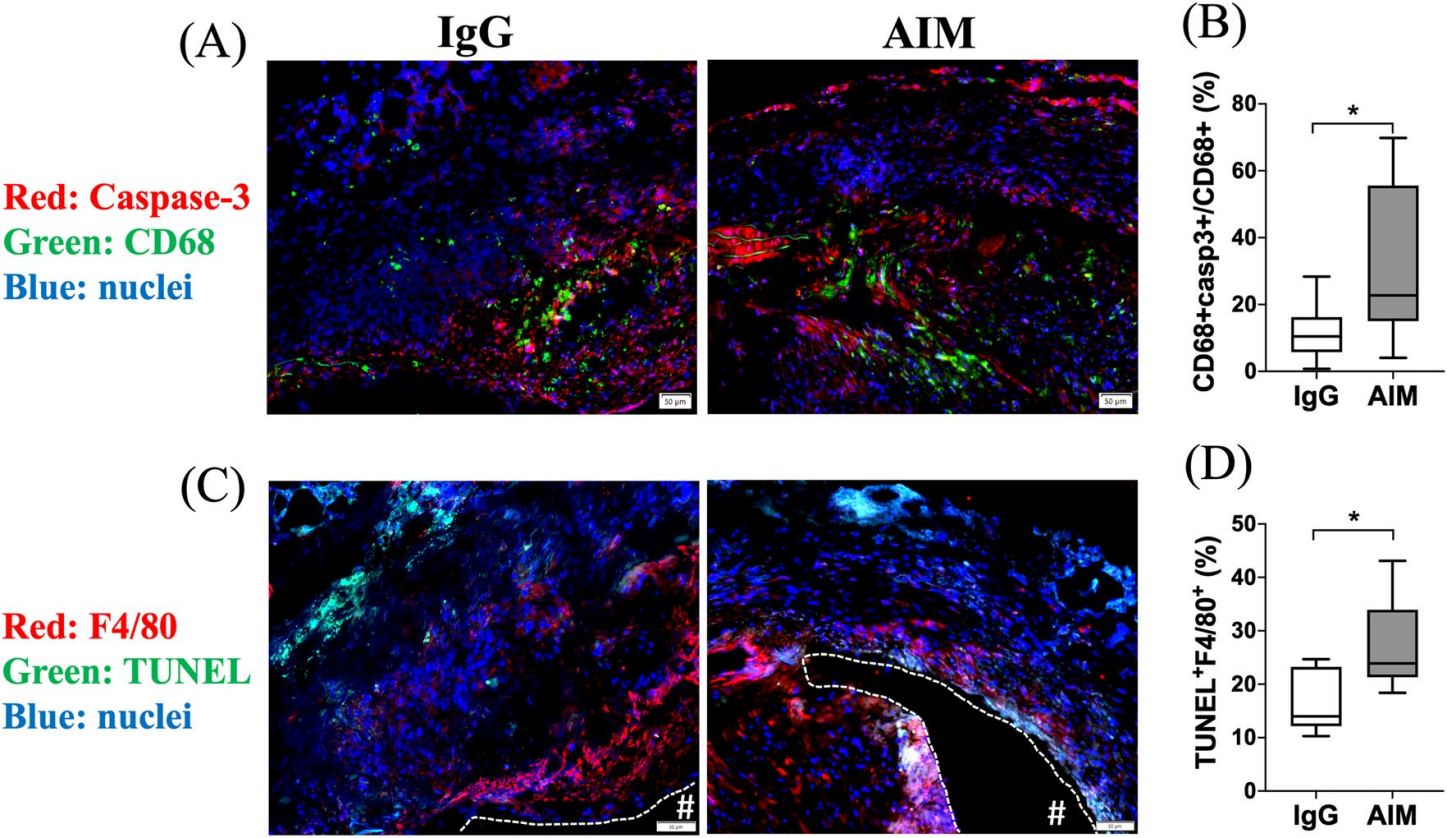
Macrophage apoptosis. (**A**) Representative images of immunofluorescence staining for apoptosis in the AIM and IgG groups. Caspase-3 (red), CD68 (green), and cell nuclei (blue). (**B**) The percentage of the CD68 + population consisting of CD68 + /caspase-3 + cells. (**C**) Representative images of immunofluorescence staining for apoptosis in the AIM and IgG groups. F4/80 (red), TUNEL (green), and cell nuclei (blue). (**D**) The percentages of cells that are TUNEL + F4/80 + . Scale bars = 50 μm. Data are means ± SEM. *p < 0.05 assessed by the Mann–Whitney *U* test. The “#” symbols enclosed by dashed lines indicate the vessel lumens.

Immunofluorescence staining was performed to illustrate the AIM distribution in the aneurysmal wall (Fig. 5A, B). The ratio of the AIM-positive area to the DAPI-positive area was significantly lower in the AIM group (Fig. 5C). The percentage of CD68^+^ cells was significantly lower in the AIM group than in the IgG group (Fig. 6C, 10.3 vs. 18.2%, respectively, p = 0.014). Staining for CD68 and iNOS showed the distribution of CD68/iNOS double-positive cells (representing M1 macrophages) in the aortic wall (Fig. 6A). The percent ratio of these double-positive cells to CD68 single-positive cells (all macrophages) was significantly lower in the AIM group than in the IgG group (Fig. 6D, 24.5 vs. 55.7%, respectively, p = 0.017). Staining for CD68 and CD206 showed the distribution of CD68/CD206 double-positive cells (M2 macrophages) (Fig. 6B), and the percent ratio of these cells to CD68 single-positive cells showed no significant difference (Fig. 6E). Immunofluorescence staining for CD68 and caspase-3 was performed to analyze macrophage apoptosis (Fig. 7A). The percentage of CD68/caspase-3 double-positive cells to CD68 single-positive cells was higher in the AIM group than in the IgG group (Fig. 7B, 22.8 vs. 10.5%, respectively, p = 0.019). In addition, macrophage apoptosis was analyzed by TUNEL assay (Fig. 7C). The percentage of TUNEL and F4/80 double-positive cells was higher in the AIM group than in the IgG group (Fig. 7D, 27.2 vs. 16.5%, respectively, p = 0.014).

## Discussion

In this study, we used a mouse model of AA induced by angiotensin (Ang) II infusion and demonstrated that intraperitoneal infusion of an anti-AIM antibody prevented AA formation and progression. We showed that the anti-AIM antibody contributed to a reduced aortic diameter at 4 weeks, but was not associated with the incidence of AA. Administration of the anti-AIM antibody decreased AIM expression in the aortic wall compared with the control group, and macrophage apoptosis via caspase-3 was promoted under the reduced AIM condition. We assumed that apoptosis selectively affected M1 rather than M2 macrophages, resulting in macrophage polarization toward the M2 phenotype. Although the anti-AIM antibody did not alter the expressions of TIMPs and MMPs, it suppressed those of most inflammatory cytokines. Consequently, elastin was better preserved qualitatively and quantitatively in the AIM group than in the control group, leading to a reduced increase in aneurysmal diameter. Among studies of AIM, this is the first to analyze elastin histologically.

Existing studies have shown that AIM is associated with lipid metabolism, immune homeostasis, and inflammatory disease control. AIM has been studied mainly for its involvement in lifestyle diseases such as obesity and metabolic syndrome, which lead to atherosclerosis and cardiovascular events^16,17^. These diseases are exacerbated by the endocytosis of AIM into adipocytes via CD36, which causes an efflux of free fatty acids induced by AIM-dependent lipolysis. Free fatty acids then stimulate chemokine production in adipocytes via activation of toll-like receptor 4 (TLR4)^13,18^. In recent years, AIM has been more broadly linked to a variety of inflammatory diseases, autoimmune diseases, tissue injuries, and infections. More specifically, these include inflammatory bowel diseases^19,20^, IgA nephropathy^21^, rheumatoid arthritis^22^, IgG4‐related disease^23^, hepatic fibrosis and cancer^24,25^, acute lung injury^26^, acute kidney injury^27^, pneumonia^28^, and sepsis^14^.

Given the association of AIM with these conditions, its regulation could significantly contribute to their treatment. Some studies of AIM-related diseases have examined the effects of neutralizing anti-AIM antibodies^14,28^. In a mouse model in which sepsis was induced by puncture of the cecum, lower mortality was achieved by intraperitoneal administration of anti-AIM antibodies than IgG antibodies^14^. Interestingly, the authors attributed this improved mortality to two different mechanisms. The first involved downregulation of inflammatory mediators, such as TNF-α and IL-6, and suppression of leukocyte infiltration. The second related to diminished release of anti-inflammatory IL-10, which prevented immune suppression and bacterial dissemination in the late phase of sepsis.

AIM also plays an important role in atherosclerosis. Studies of various cardiovascular entities demonstrated the benefits of AIM deficiency caused by genetic deletion. For example, a study using AIM knockout mice revealed a marked increase in macrophage apoptosis, accompanied by a significant reduction in the degree of atherosclerosis^15^. A study in AIM knockout mice showed attenuation of the area affected by myocardial infarction^29^. The authors suggested that AIM depletion caused a decrease in saturated free fatty acids, which reduced inflammation via the TLR-4 pathway, possibly contributing to eventual left ventricular dysfunction and remodeling. Similarly, another study in AIM knockout mice showed a reduced incidence of ventricular aneurysm rupture after myocardial infarction, caused by a decrease in M1 macrophages and subsequent decreases in MMP-2 and -9^30^. Although M1 macrophages were significantly suppressed in the AIM knockout group compared with the control group, M2 macrophages were not, which is consistent with our findings. However, our study did not reveal changes in MMP activity levels. We performed additional in vitro experiments and found no difference in *Mmp* mRNA levels in macrophages with or without anti-AIM antibody treatment (Supplementary Fig. S1). *Mmp* mRNA may decrease within 4 weeks after Ang II infusion, as shown in a study that evaluated *Mmp* mRNA using the same apolipoprotein E null mice^31^. Since MMPs are secreted by various cells besides macrophages, including fibroblasts, vascular smooth muscle cells, and leukocytes^32^, these cells might influence the levels or timings of mRNA synthesis and the enzymatic activities of MMP-2 and -9. Furthermore, considering that AIM knockout mice completely lack AIM, the molecular changes in these mice should not be directly compared to those in studies using anti-AIM antibodies. No studies thus far have examined the associations between TIMPs and AIM, so we cannot compare our results with previous ones.

Our study has several limitations. First, we administered the anti-AIM antibody intraperitoneally according to the dosing in a study on sepsis that used the same antibody for the first time^14^. To determine the frequency of anti-AIM antibody injection, we referred to our experience with biweekly injections that showed no therapeutic effect on aortic aneurysms. We therefore decided to inject antibodies weekly in this study. However, further optimization of dosing and frequency are necessary. After administration, the antibodies may migrate from the abdominal cavity into the bloodstream and then act on the aneurysm from within, or they may act directly on the retroperitoneal aneurysm from inside the peritoneum. Some reports investigated the distribution and therapeutic effects of other antibodies after intraperitoneal or intravenous injection, and found that transfer from the peritoneal cavity to blood was very rapid^33^, and there were no significant differences in therapeutic effects according to the route of administration^34^. However, the distribution of the intraperitoneally administered anti-AIM antibodies in this study remains unclear. Therefore, further studies regarding pharmacokinetic optimization are warranted. Another concerning issue related to general administration is the potential generation of antibodies against the anti-AIM antibodies following recurrent injections, which might reduce the effects. Local administration using a drug-eluting stent graft containing anti-AIM antibodies is an alternative approach that may avoid this problem if the antibodies can be incorporated into a portion of the graft that is completely isolated from the bloodstream. The second limitation is that we did not fully investigate the potential side effects of the anti-AIM antibody. AIM was initially found to suppress macrophage apoptosis, and it has been widely studied for its essential role in lipid metabolism. In this study, no adverse events related to the antibody administration were detected. While sustained, systemic administration of anti-AIM antibody could affect fatty metabolism and the immune system, this study did not include examinations, such as biochemical testing, to detect these outcomes. Third, we administered the antibody over 4 weeks, concurrently with Ang II. Since there were no cases of AA rupture in this study, we should have used a longer study period to assess whether rupture was inhibited. Fourth, another clinically important aspect of AA treatment is that therapy reduces the diameter of already-formed aneurysms. In this regard, further studies should apply our method to models in which aneurysms have already formed. Lastly, the results of our study did not suggest any theoretical role for MMPs and TIMPs even though M1 macrophages were significantly decreased. We also could not explain the mechanism whereby the anti-AIM antibody selectively suppressed M1 macrophages but did not affect M2 macrophages. Further studies are required to elucidate the molecular mechanisms involved.

## Conclusion

Our study suggested that administering the anti-AIM antibody mitigated AA progression but did not reduce its incidence. Molecular analysis demonstrated that the anti-AIM antibody induced macrophage apoptosis via caspase-3, eventually suppressing both the inflammatory response and elastin degradation in the aortic wall. Targeting AIM could serve as the foundation for developing a novel pharmaceutical therapy for AA.

## Methods

### Animals

The 24–28 week-old male apolipoprotein E-deficient mice used in this study were obtained from the Jackson Laboratory (Sacramento, CA, USA) and maintained on standard chow diets. All animals were cared for according to the Guide for the Care and Use of Laboratory Animals guidelines published by the US National Institutes of Health (Publication 85-23, National Academy Press, Washington, DC, USA, revised in 2011). All experiments and procedures in this study were approved by the Animal Experiment Advisory Committee of the Nagoya University School of Medicine (Protocol No. M210347-002), and followed the institutional ARRIVE guidelines.

### Animal model

AA was induced by subcutaneous infusion of Ang II (1000 ng/kg/min, Calbiochem, Darmstadt, Germany) for 28 days through osmotic mini-pumps (model 2004; DURECT, Cupertino, CA, USA) implanted under anesthesia with isoflurane, as previously reported^35^. Ang II-administered mice have been reported to exhibit increased systolic blood pressure^36^. In this model, AA formed in the subdiaphragmatic aorta.

### Timeline

**Figure 8:**
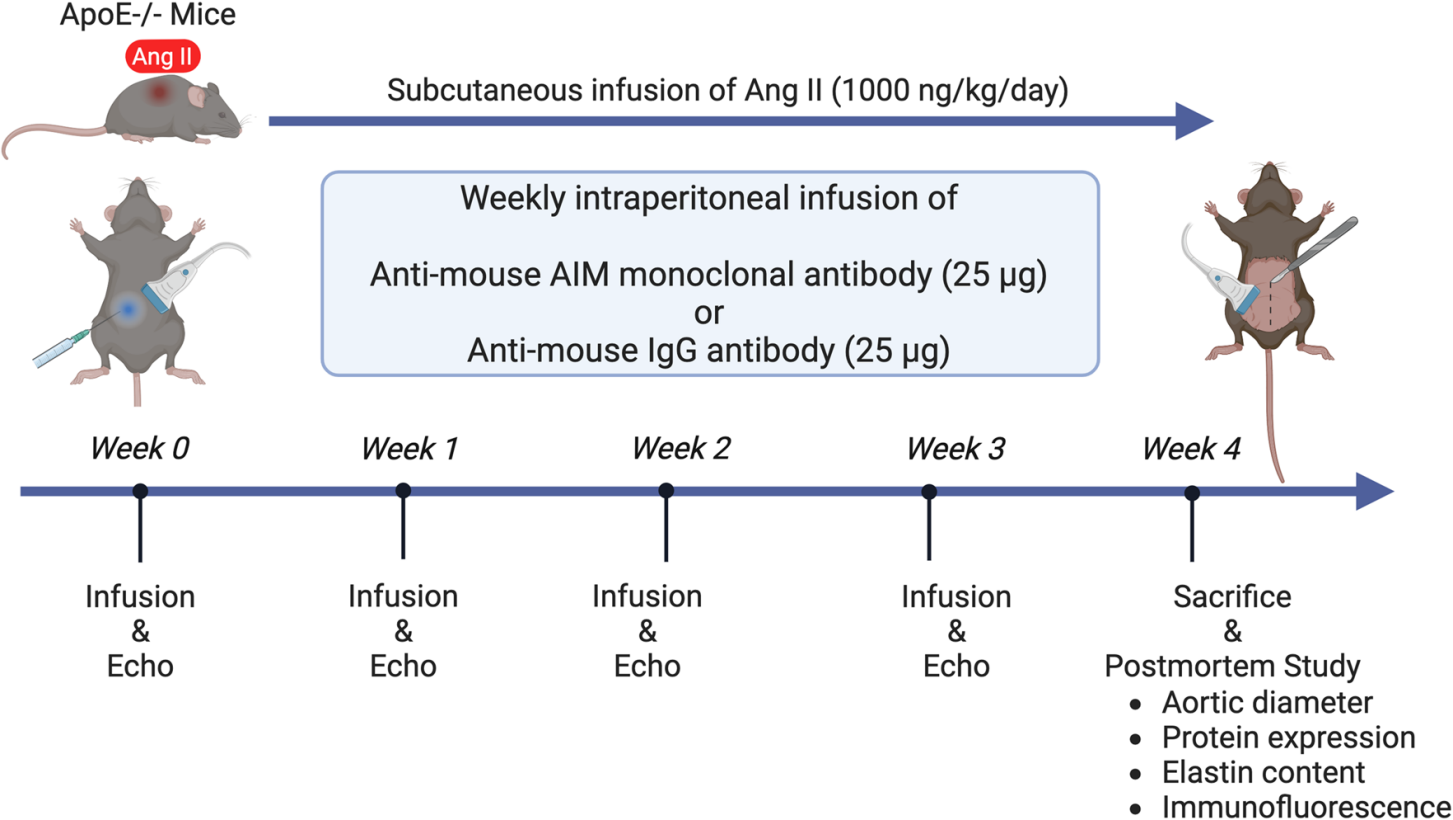
The timeline of this study. Continuous subcutaneous Ang II infusion for 28 days induced AA. Anti-mouse AIM monoclonal antibody or anti-mouse IgG antibody was injected weekly intraperitoneally. Mice of each group were sacrificed after evaluation at 0, 1, 2, and 4 weeks. Image created with BioRender.com. *AIM* apoptosis inhibitor of macrophage, *Ang* angiotensin, *ApoE* apolipoprotein E.

The timeline of this study is shown in Fig. 8. Mice were randomly assigned to anti-AIM antibody (n = 10) or IgG (n = 14) injections. Along with continuous Ang II subcutaneous infusion, anti-mouse AIM monoclonal antibody (25 µg in 0.2 ml phosphate-buffered saline (PBS), Clone No. 23B12, Trans Genic Inc., Fukuoka, Japan) or anti-mouse IgG antibody (25 µg in 0.2 ml PBS, MyBioscorce, San Diego, CA, USA) was injected intraperitoneally once a week. Echography was performed weekly after study initiation to measure the aortic diameter. Four weeks after study initiation, all animals were euthanized by isoflurane overdose. The portion of the aorta from the descending aorta to the iliac bifurcation was carefully exposed and harvested. Two-millimeter-long sections of suprarenal aorta were utilized for Elastica van Gieson (EVG) staining and immunofluorescence staining. The remaining aortas were used to assess the levels of protein expression and MMP enzymatic activity.

### Aortic diameters

Aortic diameters were recorded at 0, 1, 2, 3, and 4 weeks by echography using a LOGIQ e Premium ultrasound scanner and a 10–22 MHz probe (GE Healthcare). After harvesting, each aorta was photographed with a DP70 digital camera (Olympus, Tokyo, Japan) attached to a stereomicroscope, used with DP controller software (v.03.021, Olympus, https://www.olympus-lifescience.com/en/support/downloads/dp-bsw_ver0302_step/), and was measured with digital image analysis software (ImageJ v.1.54g, National Institutes of Health, Bethesda, MD, USA, https://imagej.net/ij/) by calibration with ruler correction. AA formation was defined as at least a 1.5-fold increase in aortic diameter relative to the original diameter, according to well-known diagnostic criteria^37^. In addition, a less than 1.3-fold increase relative to the original diameter, without typical AA features such as arteriosclerosis, calcification, or plaque, was considered to indicate failure of AA formation.

### EVG staining

Each harvested suprarenal aorta was embedded in optimal cutting temperature compound (Leica Microsystems, Buffalo Grove, IL, USA) and sliced with a microtome cryostat (Leica Microsystems). The cross-Sects. (10 μm thickness) were stained with Weigert’s resorcin–fuchsin (Muto Pure Chemicals, Tokyo, Japan). Each slide was photographed with a DP80 digital camera (Olympus) attached to a stereomicroscope, used with DP controller software (Olympus). Photographic images were obtained (Olympus). Images were then analyzed using Cellsens software (v.2.3, Olympus, https://www.olympus-lifescience.com/en/support/downloads/) to determine the area of elastin staining as the percent area of elastin and the percent area of the medial components between the elastic lamellae (elastin gap area), both compared with the total medial tissue area, and to identify the number of elastic lamellae and the number of elastin breaks. The elastic lamellae were assessed for the average number of elastic lamellae and the total number of breaks per elastic lamina by counting them circumferentially in all lamina. Experiments included 10 mice per group, using three consecutive aortic sections from each animal as previously described^38^.

### Enzyme-linked immunosorbent assay

AA tissues were homogenized using RIPA buffer (Fujifilm Wako Pure Chemical Corporation, Osaka, Japan). Lysate protein concentrations were measured with a Qubit protein assay kit and Qubit 2.0 fluorometer (Thermo Fisher Scientific, Waltham, MA, USA). For each enzyme-linked immunosorbent assay (ELISA) kit (AIM, IL-1β, IL-6, TNF-α, tissue inhibitor of metalloproteinases (TIMP)-1, and MCP-1: Invitrogen, Carlsbad CA, USA; TIMP-2: Abcam, Cambridge, MA, USA; Caspase-3: Cusabio, Houston, TX, USA), an equal concentration of total protein was applied and then detected.

### Enzymatic activities of MMP-2 and -9

Endogenously active MMP-2 and -9 in aortic tissues were measured using a SensoLyte 520 MMP-2 assay kit (ANASPEC, Fremont, CA, USA) and a SensoLyte 520 MMP-9 activity assay kit (ANASPEC), respectively. An equal concentration of total protein was applied to each assay kit and then detected.

### Immunofluorescence staining

Frozen, 10 μm-thick cross-sections were fixed with 4% paraformaldehyde for 15 min, and blocking was performed with 10% bovine serum albumin for 30 min. Then, antigen retrieval was performed with HistVT One (Nacalai Tesque, Kyoto, Japan) at 70 ℃ for 20 min. To determine AIM distribution, the primary antibody was a rabbit anti-AIM antibody (1:50, AVIVA Systems Biology, San Diego, CA, USA). The secondary antibody was an anti-rabbit IgG Alexa Fluor 555–conjugated antibody (1:5000, Cell Signaling Technology, Danvers, MA, USA) bound to the anti-AIM antibody. Apoptotic macrophages were detected by the TUNEL method, using an in situ apoptosis detection kit (Takara, Shiga, Japan). The primary antibodies were a rat anti-CD68–Alexa Fluor 488 conjugate (1:250, Cell Signaling Technology), a rat anti-F4/80 antibody (1:200, Bio-Rad Laboratories, Hercules, CA, USA) as pan-macrophage markers, and a rabbit anti-caspase-3 antibody (1:200, GeneTex, Irvin, CA, USA) as a marker for apoptosis. The secondary antibodies were an anti-rat IgG Alexa Fluor 555–conjugated antibody (1:1000, Cell Signaling Technology) bound to the anti-F4/80 antibody, and an anti-rabbit IgG Alexa Fluor 555–conjugated antibody (1:5000, Cell Signaling Technology) bound to the anti-caspase-3 antibody. For macrophage phenotype analysis, the primary antibodies were a rat anti-CD68–Alexa Fluor 488 conjugate and a rabbit anti-inducible nitric oxide synthase (iNOS)–Alexa Fluor 555 conjugate (1:50, Abcam, Cambridge, MA, USA) for M1 macrophages, and a rat anti-CD68–Alexa Fluor 488 conjugate and a rabbit anti-CD206-Alexa Fluor 594 conjugate (1:50, Cell Signaling Technology) for M2 macrophages. In all slides, cell nuclei were stained with DAPI Fluoromount-G (Southern Biotech, Birmingham, AL, USA). Negative control experiments used rat IgG and rabbit IgG isotype control antibodies (Cell Signaling Technology) at the same concentration as the primary antibody. Slides were photographed with a DP80 digital camera attached to a stereomicroscope, used with DP controller software (Olympus). Fluorescence signals were quantified in the entire aortic wall, including the lumen, media, and adventitia, using cellSence software ver. 2.3 (Olympus). Fluorescence signals were quantified in the entire aortic wall, including the lumen, media, and adventitia.

### Statistical analysis

The Mann–Whitney *U* test was used in elastin, ELISA, MMP, and immunofluorescence staining analyses. Kaplan–Meier analyses followed by log-rank tests were performed for survival rate. Longitudinal changes in aortic diameter were analyzed using two-way repeated measure ANOVA followed by Tukey’s multiple comparisons test. Fisher’s exact test was applied for AA incidence. All statistical analyses were conducted using GraphPad Prism for Mac (version 8, San Diego, CA, USA). Data are expressed as mean ± standard error of the mean (SEM). p-values < 0.05 were considered significantly different.

### Supplementary Information

Supplementary Information.

## Data Availability

The data will be shared on a request basis. Please directly contact the corresponding author to request data sharing.
