# Supplementary material for: Administration of an antibody against apoptosis inhibitor of macrophage prevents aortic aneurysm progression in mice
Source: Sci Rep. 2024 Jul 10;14:15878. doi: 10.1038/s41598-024-66791-7 (PMC11233551; doi:10.1038/s41598-024-66791-7)
Supplement: Supplementary file 1 — Supplementary Information. [file 41598_2024_66791_MOESM1_ESM.docx]

**SUPPLEMENTARY INFORMATION**

Administration of an Antibody against Apoptosis Inhibitor of Macrophage Prevents Aortic Aneurysm Progression in Mice

Taro Fujii†, MD; Aika Yamawaki-Ogata†, PhD; Sachie Terazawa, MD, PhD; Yuji Narita*, MD, PhD; Masato Mutsuga, MD, PhD

Department of Cardiac Surgery, Nagoya University Graduate School of Medicine

65 Tsurumaicho Showa Nagoya Aichi, 466-8550 Japan

†Equal contributors

* Correspondence: Yuji Narita, ynarita@med.nagoya-u.ac.jp

65 Tsurumaicho Showa Nagoya Aichi, 466-8550 Japan

**Supplementary Methods**

**Pretreatment of cultured macrophages with anti-AIM antibody and Quantitative RT-PCR**

Cell culture and expansion of murine macrophages were performed as previously described.^38^ Macrophages were plated at 2 × 10^4^ cells per well in a 96-well plate, and pretreated with rSLPI (R&D Systems) at doses of 0 and 1 μg/mL with incubation at 37°C in a humidified atmosphere of 5% CO_2_ in air for 24 h. The medium was then replaced for 24 h with growth medium containing 10 ng/mL liposaccharide (LPS, SIGMA-Aldrich) and 2 ng/mL TNF-α (recombinant human, Peprotech, Cranbury, NJ, USA). After incubation, cells underwent RNA extraction (n = 4, respectively).

Total cellular RNA was extracted from M1 macrophages with the NucleoSpin RNA kit (Macherey–Nagel GmbH & Co. KG, Düren, Germany). Samples were normalized to 0.1 μg of total RNA. Then, cDNA was synthesized using the Takara PrimeScript RT reagent kit (Takara Bio Inc., Shiga, Japan). Quantitative real-time polymerase chain reaction (PCR) was performed using a thermal cycler (CFX connect, Bio-Rad Laboratories, Hercules, CA, USA) based on the standard curve using Thunderbird SYBR qPCR Mix (TOYOBO, Osaka, Japan). The PCR cycling conditions were as follows: 95 °C for 1 min and then 40 cycles at 95 °C for 15 s for denaturation, followed by 60 °C for 45 s for annealing. To amplify macrophage genes, we selected primers for MMP-2 and -9, along with β-actin (Sigma-Aldrich) as a control (Supplementary Table 1). All data were analyzed by CFX Maestro ver.1.1 Software (Bio-Rad).

| **Gene** | **Accession number** | **Sequences of forward and reverse primers** |
| --- | --- | --- |
| *Actb* | NM_007393 | 5′–AGAGGGAAATCGTGCGTGAC–3′ |
|  |  | 5′–CAATAGTGATGACCTGGCCGT–3′ |
| *Mmp-2* | NM_008610 | 5′–GTCGCCCCTAAAACAGACAA–3′ |
|  |  | 5′–GGTCTCGATGGTGTTCTGGT–3′ |
| *Mmp-9* | NM_013599 | 5′–CGTCGTGATCCCCACTTACT–3′ |
|  |  | 5′–AACACACAGGGTTTGCCTTC–3′ |

Supplementary Table 1. Primer sequences.

**Supplementary Results**

**Anti-AIM antibody does not influence *Mmp-9* mRNA levels in macrophages**

Supplementary Fig. S1 shows the mRNA expression levels in macrophages pretreated with anti-AIM antibody or non-pretreated. *Mmp-2* mRNA was below detection limits in both groups, and *Mmp-9* mRNA levels showed no significant difference between the groups.


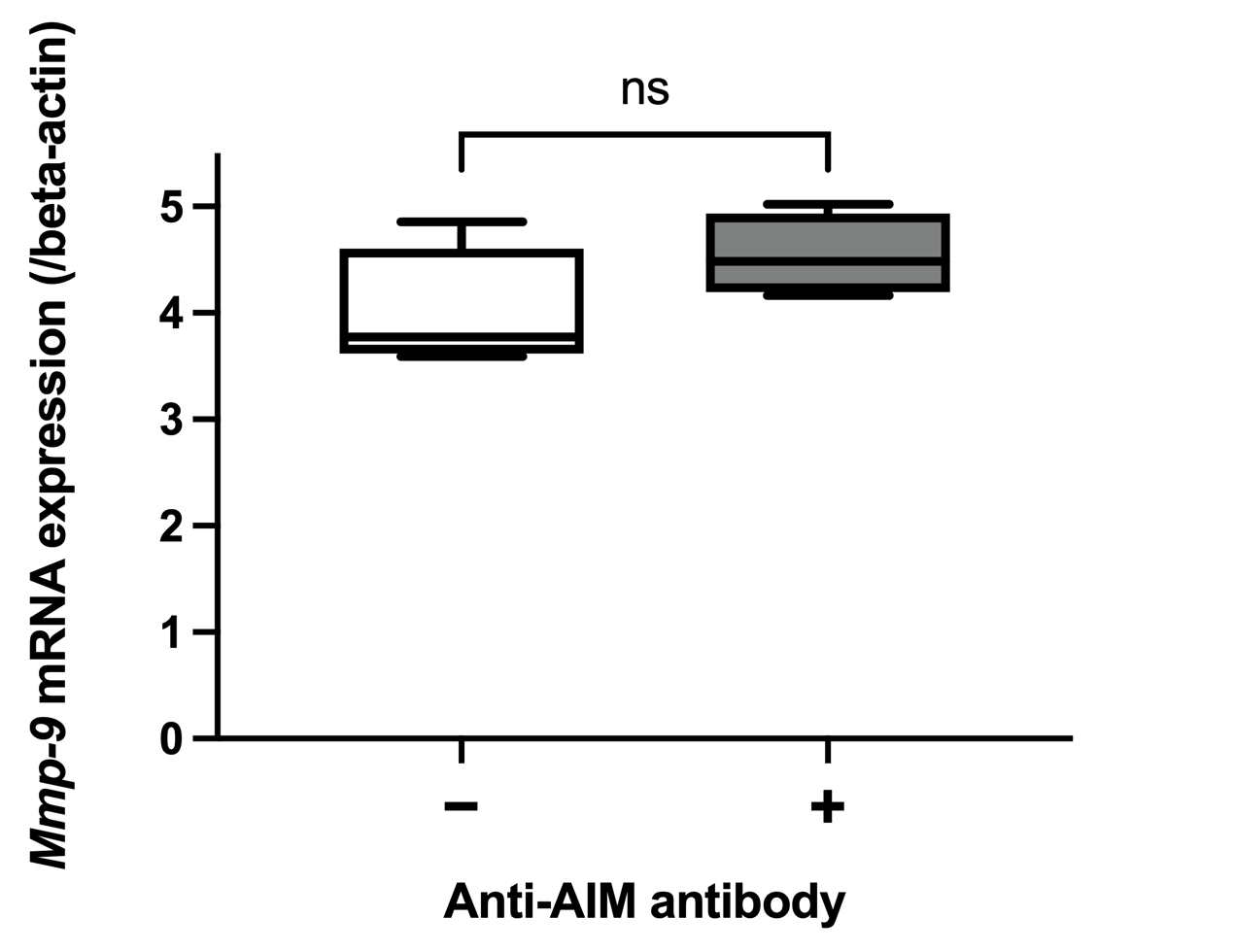


**Supplementary Fig. S1**. ***Mmp-9* mRNA expression levels**

In vitro quantitative analysis of *Mmp-9* mRNA in macrophages treated with anti-AIM antibodies. Data are means ± SEM and assessed by student t-test. ns: not significant.

**Magnified immunofluorescence staining distinguishes between M1 and M2 macrophages**

Supplementary Fig. S2 shows the distinction between pro-inflammatory “M1 macrophages” and anti-inflammatory “M2 macrophages”.


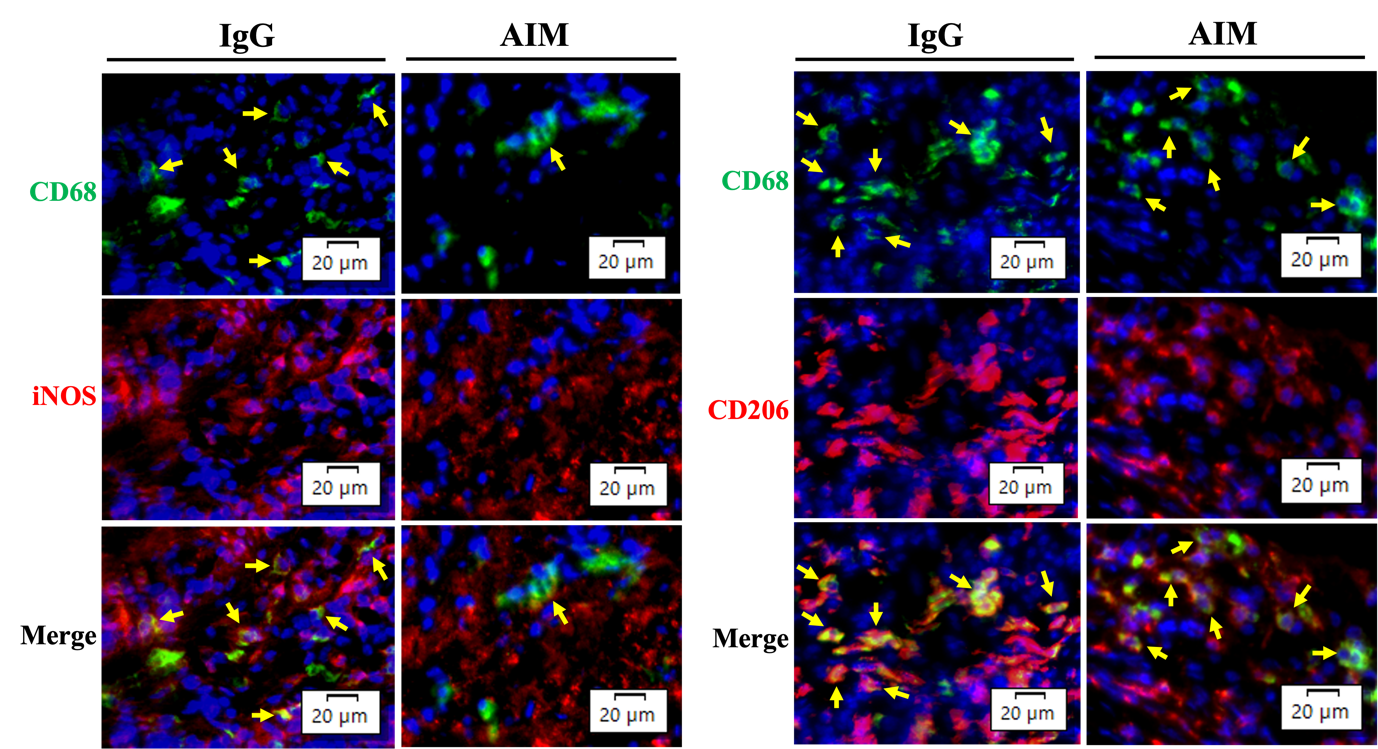


**Supplementary Fig. S2**. **Immunofluorescence staining for CD68 and iNOS or CD206**

These images are the magnified images of Fig. 6. The yellow arrows indicate the macrophages co-staining positively for CD68 (green) and iNOS (red) or CD206 (red).
